# Skin microbiome and cutaneous aging mechanisms and clinical implications

**DOI:** 10.3389/fmicb.2026.1917816

**Published:** 2026-08-20

**Authors:** Jhommara Bautista, Andrea Iñiguez-Ramírez, Jaime Andrés Villegas-Chávez, Doménica Bunces-Larco, Andrés López-Cortés

**Affiliations:** 1 Cancer Research Group (CRG), Faculty of Medicine, Universidad de Las Américas, Quito, Ecuador; 2 Consultant, Quito, Ecuador; 3 Esticca Medical Center, Medicina Estética Especializada, Quito, Ecuador

**Keywords:** age-associated microbial variation, aging, homeostasis, inflammation, oxidative stress, skin microbiome

## Abstract

The skin microbiome is an integral component of the cutaneous ecosystem and contributes to barrier, immune, and metabolic homeostasis. Available evidence is examined across three distinct levels: microbial community structure, functional activity, and host biological response. This ecological–functional distinction is necessary because taxonomic abundance alone does not establish microbial activity, biological effects, or causality. Age-associated microbial variation is considered within the physiological context of cutaneous aging, including reduced sebaceous activity, altered hydration and surface pH, impaired barrier recovery, chronic low-grade inflammation, oxidative stress, and extracellular matrix deterioration. Microbial alterations reported in acne, atopic dermatitis, and rosacea further illustrate how changes in the cutaneous environment can modify host–microbiome interactions and contribute to clinically relevant phenotypes. Translational developments in dermatology and aesthetic medicine include microbiome-compatible skincare, prebiotic and postbiotic formulations, live biotherapeutic approaches, and strategies intended to preserve microbial and barrier recovery after dermatological procedures. Interpretation of the available literature remains limited by low microbial biomass, anatomical and interpersonal heterogeneity, contamination risk, differences in sampling and sequencing methods, and limited functional and longitudinal resolution. Observed microbial remodeling should be interpreted within a bidirectional host–microbiome relationship. Physiological changes that accompany aging can reshape microbial ecology, whereas microbial products may modify barrier and immune responses. Current evidence does not establish whether these microbial alterations are causes, consequences, or correlates of cutaneous aging. Integration of strain-resolved microbiome data with microbial gene expression, metabolite measurements, host molecular responses, and clinical phenotypes will be required to identify biologically relevant microbial functions and determine their value in dermatological practice.

## Introduction

Cutaneous aging represents a major biological and clinical challenge, reflecting the cumulative impact of intrinsic determinants, including chronological aging and genetic background, together with extrinsic exposures involving environmental and lifestyle influences (Wang et al., 2025). Progressive changes alter structural organization and physiological function in a tissue that operates as the primary interface between the organism and the external environment. A multilayered architecture composed of diverse cell populations supports barrier integrity and environmental interaction, while simultaneously accommodating a complex consortium of microorganisms distributed across ecological niches defined by physicochemical parameters such as pH, moisture, and sebum content. Continuous exposure to environmental stressors, together with intrinsic biological aging, contributes to cumulative alterations that reshape both tissue structure and surface conditions (Buck et al., 2026; Howard et al., 2022).

Age-associated variation involves modifications in tissue architecture, cellular composition, and microenvironmental stability, leading to reduced functional capacity and altered visible characteristics. Parallel changes in physiological parameters, including moisture, lipid composition, and barrier properties, modify local ecological conditions that define microbial colonization patterns. Coordinated variation across epidermal, dermal, and surface compartments determines the state of the cutaneous environment, while spatial heterogeneity across anatomical regions generates distinct ecological niches that support diverse microbial assemblages (Moissl-Eichinger et al., 2017; MacGibeny et al., 2025).

Microbiota vary across the lifespan, with differences in diversity, abundance, and structure associated with age and host physiological state. Early colonization patterns evolve over time into site-specific configurations shaped by host and environmental factors, indicating that microbial community assembly represents a dynamic process influenced by host biology throughout life. Across adulthood, variation in physiological conditions is associated with differences in composition across age groups and anatomical locations. Overall microbial structure reflects underlying host-related and environmental variation (Kim et al., 2022; He et al., 2025).

Research addressing microbiome dynamics in aging remains predominantly descriptive and fragmented, with limited integration between microbial profiling, functional measurements, and host biological context. Sequencing-based studies have characterized microbial composition across anatomical sites, populations, and age groups, with emphasis on taxonomic diversity and distribution (Prescott et al., 2017). Associations between microbial patterns and host-related variables, including age, physiology, and environmental exposure, have been consistently reported. However, biological interpretation requires integration of microbial composition with functional outputs, host phenotypes, and local microenvironmental characteristics. Uncertainty regarding the functional role of microbial communities within the broader cutaneous system, together with methodological heterogeneity and limited standardization, continues to constrain interpretation and cross-study comparability (Wyles et al., 2025; Cheng et al., 2024; Fernández-Carro et al., 2025).

Resident microbiota constitute a component of the cutaneous microenvironment, coexisting with structural elements, resident cells, and environmental inputs that collectively define the organization of the skin ecosystem. Associations between microbial features and phenotypic characteristics are reported across independent studies. Integration of microbiome data with structural and physiological parameters is required to contextualize microbial variation within cutaneous biology (Shibagaki et al., 2017).

Recent reviews have summarized age-associated microbial patterns, microbiome–host mechanisms, and microbiome-directed cosmetic or dermatologic interventions (Ratanapokasatit et al., 2022; Suri et al., 2025; Taléns-Visconti et al., 2026). However, uncertainty remains regarding how these observations should be interpreted across ecological, functional, and causal levels. Taxonomic differences are frequently reported without establishing whether they reflect altered substrate availability, strain-level variation, active microbial pathways, measurable metabolite production, or downstream effects on host tissues.

**Figure 1:**
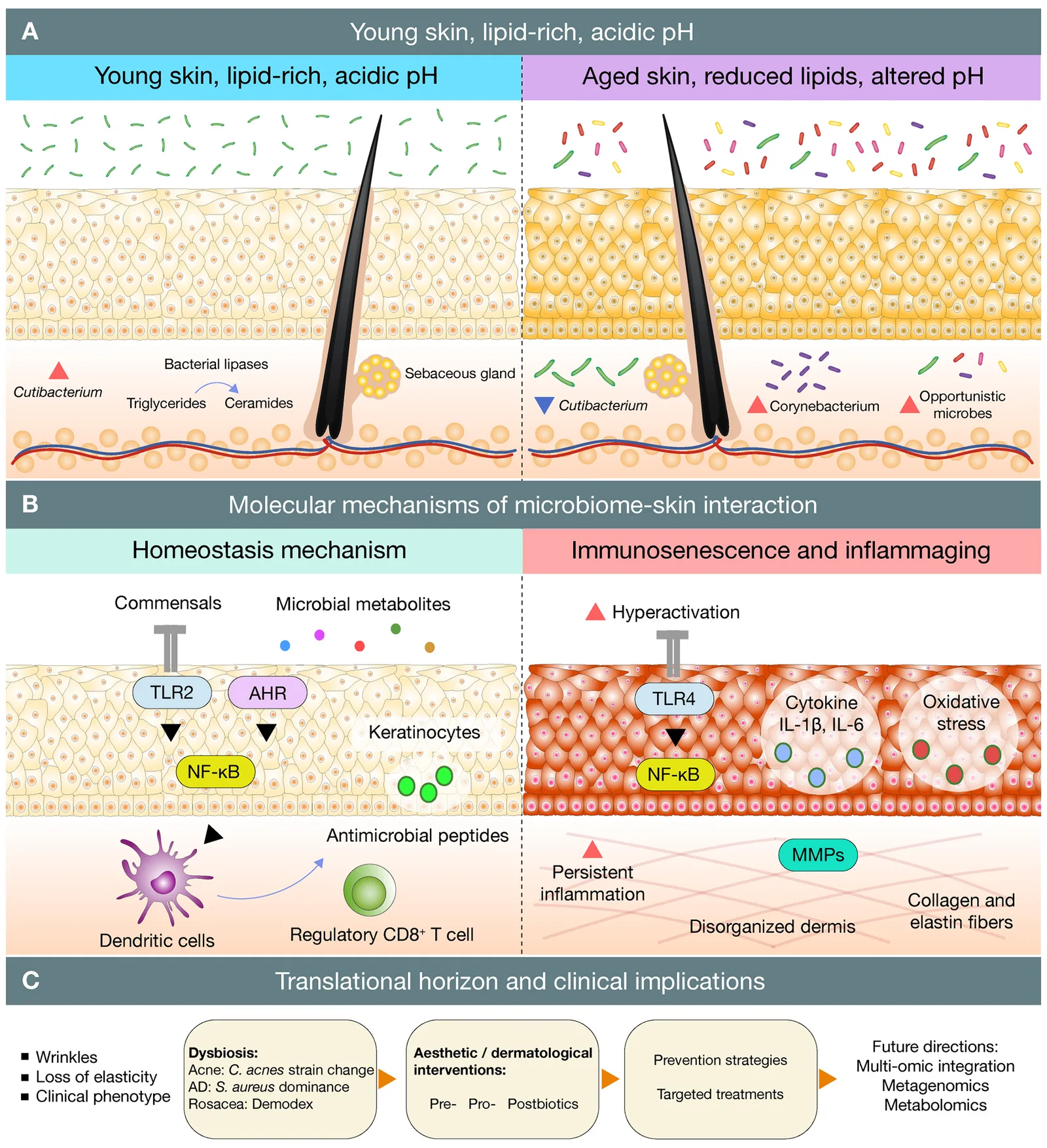
The microbiome–skin axis in aging, homeostasis, and dermatological interventions. The microbiome–skin axis in aging, homeostasis, and dermatological interventions. This figure summarizes ecological, molecular, and translational interactions linking the skin microbiome with cutaneous homeostasis, aging biology, and dermatological applications. **(A)** In younger skin, lipid-rich and acidic conditions support microbial functions related to lipid hydrolysis, fermentation, organic acid production, and sphingolipid metabolism. Age-associated reductions in sebum, changes in hydration and surface pH, impaired barrier recovery, and immune senescence alter microbial community composition, strain distribution, substrate availability, and metabolic activity. Variations in *Cutibacterium, Corynebacterium*, and other taxa are presented as ecological indicators of this functional remodeling rather than as independent drivers of cutaneous aging. **(B)** Signals derived from commensal microorganisms can influence TLR2, AHR, and NF-κB signaling, antimicrobial peptide production, barrier integrity, and immune homeostasis. In aged skin, microbial functional remodeling may shift the balance between barrier-supportive metabolites and pro-inflammatory microbial ligands. Experimental studies indicate that microbiome-derived signals can modulate AHR, PRR–NF-κB/MAPK, cytokine, and matrix metalloproteinase pathways. However, a direct causal relationship between age-associated microbial alterations and dermal matrix degradation has not been demonstrated in humans. **(C)** Microbial and host changes occur alongside wrinkles, loss of elasticity, reduced barrier resilience, and microbiome-associated dermatological conditions, including acne, atopic dermatitis, and rosacea. Strategies based on prebiotics, probiotics, postbiotics, and other microbiome-supportive interventions may inform future approaches in precision dermatology and aesthetic medicine.

To address this gap, the review examines age-associated microbiome remodeling within the physiological context of aging skin. Evidence is organized from anatomical niches and local physicochemical conditions to community and strain structure, microbial functional outputs, host signaling, aging phenotypes, and clinical translation. Changes in *Cutibacterium, Corynebacterium*, and other taxa are assessed as ecological indicators, plausible functional contributors, or consequences of altered sebum production, hydration, surface pH, barrier recovery, immune senescence, oxidative stress, and extracellular matrix homeostasis. Particular attention is given to distinguishing taxonomic abundance and encoded functional potential from pathway activity, metabolite production, host response, and causal evidence. The resulting framework integrates microbial ecology, aging biology, functional microbiomics, and dermatologic translation while specifying the evidence required to support biological interpretation and clinical application (Figure 1).

## Skin microbiome architecture and ecological niches

Human skin contains a spatially heterogeneous microbiome structured by anatomical location and local physicochemical conditions. Microbial composition varies systematically across body sites, forming a reproducible biogeographic pattern in which sebaceous, moist, and dry regions support distinct microbial communities. Foundational sequencing and metagenomic studies established that local skin physiology is a major determinant of both taxonomic composition and functional capacity, while individual-specific microbial features contribute an additional level of variation (Pérez-Losada and Crandall, 2023; Byrd et al., 2018; Oh et al., 2014). Communities from the same anatomical niche are therefore generally more similar than communities from regions with different physiological properties.

Lipid-rich sites favor microorganisms capable of using sebaceous triglycerides, fatty acids, squalene, and other follicular substrates. Humid regions support traits related to sweat-derived nutrient utilization, osmotic adaptation, adhesion, and intermicrobial competition. Dry sites often contain more taxonomically diverse and variable communities, although biomass is usually lower because water and nutrients are limited. *Cutibacterium, Staphylococcus, Corynebacterium*, and *Malassezia* remain useful ecological indicators of these environments, but their relative abundance does not by itself define their effect on the host. Biological activity depends on strain-specific gene content, pathway expression under local conditions, and substrate availability (Oh et al., 2014; Suri et al., 2025).

A core skin microbiome may be defined at both taxonomic and functional levels. The taxonomic core includes microorganisms recurrently detected within particular anatomical niches or across defined populations. The functional core comprises conserved activities involved in lipid hydrolysis, amino acid metabolism, organic acid production, colonization resistance, stress tolerance, and modulation of epidermal signaling. Functional redundancy permits taxonomically distinct microorganisms to perform comparable biochemical activities. Conversely, strains belonging to the same species may differ in gene content, metabolic output, antimicrobial capacity, and inflammatory potential. Interpretation based only on community composition may therefore overlook biologically relevant functional and strain-level differences (MacGibeny et al., 2025; Park et al., 2026).

Sebum, moisture, pH, temperature, and oxygen availability further regulate the expression and activity of microbial pathways within each niche. Sebaceous substrates support lipid-processing functions, whereas acidic conditions restrict poorly adapted microorganisms and contribute to community stability. Sweat secretion modifies nutrient availability and ionic conditions in humid sites, while reduced moisture limits microbial growth and metabolic activity in dry regions. These local properties influence not only which microorganisms are present but also which microbial functions are biologically active at the skin surface (Swaney et al., 2023).

Environmental exposures and lifestyle factors further modify this spatial organization. Ultraviolet radiation, climate, hygiene practices, and cosmetic use can alter surface lipids, hydration, pH, barrier integrity, and nutrient availability, with corresponding effects on microbial composition and activity. Age-related physiological changes operate through many of the same ecological variables by modifying the conditions that favor particular strains and metabolic pathways. Age-associated microbial patterns should therefore be examined in relation to changes in the cutaneous environment rather than interpreted as isolated taxonomic shifts (Maloney et al., 2025; Kim et al., 2021; Dimitriu et al., 2019).

## Functional outputs of the skin microbiome

The skin microbiome generates a defined repertoire of biochemical products through transformation of host-derived lipids and amino acids located in the stratum corneum and sebaceous secretions. Sebaceous glands release triglycerides, wax esters, squalene, and cholesterol onto the skin surface, which are subsequently processed by microbial enzymes. Bacterial and fungal lipases hydrolyze triglycerides into free fatty acids and glycerol, providing substrates for downstream metabolic reactions and sustaining microbial growth in a nutrient-limited environment. Continuous degradation of complex lipids maintains a pool of low-molecular-weight compounds that defines the chemical composition of the skin surface (Kengmo Tchoupa et al., 2023; Raza et al., 2025).

Short-chain fatty acids (SCFAs) arise from fermentation of lipid-derived substrates, particularly glycerol and fatty acids released from sebum. Cutaneous bacteria adapted to sebaceous niches, including *Cutibacterium acnes*, convert available substrates into acetate, propionate, and butyrate. Propionate constitutes a dominant metabolic product in lipid-rich environments associated with the pilosebaceous unit. Accumulation of SCFAs contributes to the free fatty acid fraction detected on the skin surface and sustains an acidic chemical profile that characterizes the cutaneous environment. Additional organic acids, including acetate and valerate, are produced through related fermentation pathways, expanding the diversity of small metabolites present at the skin interface (Flowers and Grice, 2020).

Microbial metabolism of host lipids generates a broad range of lipid derivatives beyond SCFAs. Hydrolysis of phospholipids and sphingolipids releases fatty acids and intermediate lipid species that undergo further transformation into structurally diverse compounds. Sphingomyelin cleavage mediated by microbial enzymes produces ceramides derived from membrane sphingolipids, which represent a major fraction of epidermal lipids (Li Z. et al., 2026; Qiu et al., 2022; Mercer et al., 2025). In addition to modifying existing lipid pools, certain commensal microorganisms contribute directly to ceramide generation. *Staphylococcus epidermidis* produces a sphingomyelinase capable of converting host sphingomyelin into ceramides within the stratum corneum. Experimental studies have shown that microbial sphingomyelinase activity can increase cutaneous ceramide content and reduce transepidermal water loss under conditions of barrier disruption, supporting a contribution of microbial lipid metabolism to epidermal barrier homeostasis (Zheng et al., 2022). Polyunsaturated fatty acids originating from sebum are converted into oxylipins through oxidation processes, yielding oxygenated lipid mediators detectable on the skin surface. Variation in oxylipin profiles correlates with microbial abundance and sebaceous activity, indicating an association between microbial metabolism and lipid-derived outputs (Pagac et al., 2025).

Fungal taxa, particularly *Malassezia*, exhibit extensive lipid catabolic capacity supported by multiple lipases and phospholipases, enabling efficient utilization of host-derived lipids and generation of secondary lipid compounds. Lipid degradation by fungal and bacterial communities produces additional molecules, including antimicrobial fatty acids and derivatives such as azelaic acid and oleic acid, which arise from transformation of sebaceous substrates. Expansion of lipid-derived outputs reflects the diversity of enzymatic activities encoded within the skin microbiome (Nguyen and Kalan, 2022; Mercer et al., 2025).

Amino acid metabolism provides an additional source of microbial products. Skin-associated microorganisms transform tryptophan into indolic compounds that can participate in communication with epidermal cells. Experimental studies using defined microbial communities indicate that commensal-derived tryptophan metabolites promote epidermal differentiation and strengthen barrier function, supporting a direct functional relationship between microbial metabolism and cutaneous homeostasis (Uberoi et al., 2025). Additional products derived from amino acid catabolism include phenylacetic acid and porphyrins, generated through bacterial degradation of phenylalanine and δ-aminolevulinic acid, respectively (Zhou et al., 2026; Uberoi et al., 2025; Tang et al., 2025).

The functional output of the skin microbiome is closely linked to its enzymatic capacity. Bacterial and fungal communities express lipases, phospholipases, proteases, and sphingomyelinases that enable degradation of host lipids and proteins into utilizable substrates. Enzymatic activity is required to mobilize nutrients embedded within lamellar lipid membranes, where substrate accessibility remains restricted. Microbial surfactant molecules and lipopeptides facilitate solubilization of hydrophobic compounds, supporting continued lipid utilization and metabolite generation (Pagac et al., 2025; Smythe and Wilkinson, 2023).

Microbial metabolites contribute directly to the chemical environment and structural organization of the skin surface. Free fatty acids and other organic acids help preserve the acidic pH of the stratum corneum, limiting the growth of microorganisms poorly adapted to cutaneous conditions and favoring the persistence of resident taxa. Microbial modification of host-derived lipids may further alter the composition and organization of the stratum corneum lipid matrix, whereas metabolites generated from amino acids participate in local chemical signaling. Through such activities, the skin microbiota contributes to colonization resistance, epidermal differentiation, and barrier integrity. The magnitude and direction of these effects depend on the taxa present, their metabolic activity, and site-specific features such as pH, moisture, and sebaceous lipid availability (Mercer et al., 2025; Flowers and Grice, 2020; Harris-Tryon and Grice, 2022).

## Molecular mechanisms of microbiome–skin interaction

Cutaneous microbiota are sensed by keratinocytes and resident immune cells through pattern recognition receptors, predominantly TLR2 and TLR4, which recognize conserved microbial ligands and initiate innate immune activation. TLR2 detects Gram-positive bacterial components, including lipoproteins from commensal species, particularly *Staphylococcus epidermidis*, promoting antimicrobial peptide production and maintaining basal immune surveillance. TLR4 recognizes lipopolysaccharides from Gram-negative bacteria and induces pro-inflammatory signaling that modulates local immune responses (Li et al., 2025b; Cha et al., 2025). Continuous PRR engagement by commensal microbiota maintains immune calibration at the epidermal interface, enabling rapid responsiveness while preventing excessive inflammatory activation.

TLR2 signaling proceeds predominantly through MyD88-dependent pathways, whereas TLR4 engages both MyD88-dependent and TRIF-dependent signaling. The MyD88-dependent branch activates the IKK complex, promoting NF-κB nuclear translocation, and also stimulates ERK1/2, JNK, and p38 MAPK, which regulate AP-1 complexes containing c-Jun and c-Fos. Depending on the ligand and cellular context, NF-κB and AP-1 regulate the expression of cytokines, chemokines, antimicrobial peptides, redox-response mediators, and matrix metalloproteinases. Dysregulated microbiota–TLR signaling may therefore enhance inflammatory transcriptional programs, including IL1B and IL6 expression (Szabó et al., 2025; Zhang et al., 2025). Microbiota-derived ligands and metabolites can also activate AHR-dependent transcriptional programs in keratinocytes. Experimental models demonstrate that commensal microbiota support epidermal differentiation, barrier integrity, and repair through keratinocyte AHR signaling, providing a direct mechanistic link between microbial activity and cutaneous barrier function (Uberoi et al., 2021).

Inflammasome signaling links microbial exposure with cutaneous inflammation. TLR–NF-κB signaling can prime the pathway by increasing NLRP3 and pro-IL-1β expression, whereas potassium efflux, oxidative stress, and membrane damage can promote formation of the NLRP3–ASC–caspase-1 complex and the maturation of IL-1β and IL-18. In a keratinocyte–sebocyte coculture model, porphyrins produced by an acne-associated Cutibacterium acnes strain induced potassium efflux, NLRP3 inflammasome assembly, and IL-1β release. A strain more commonly detected in healthy skin produced a weaker response. The findings demonstrate strain-dependent inflammasome activity under acne-related experimental conditions but do not show that age-associated microbiome alterations activate NLRP3 in human skin (Spittaels et al., 2021).

Keratinocytes operate as central signaling nodes that translate microbial detection into coordinated immune outputs. Following PRR activation, keratinocytes release cytokines and chemokines that regulate recruitment and activation of dermal immune populations, including dendritic cells and T lymphocytes. Microbial-derived metabolites modulate keratinocyte signaling thresholds, influencing cytokine output and downstream immune activation. Reduced availability of microbiota-derived metabolites correlates with increased inflammatory mediator expression and enhanced matrix-degrading activity, indicating direct control of inflammatory signaling intensity. In parallel, microbiota-derived ligands activate intracellular receptors, particularly the aryl hydrocarbon receptor (AHR), in keratinocytes, linking microbial sensing to transcriptional programs that integrate immune signaling with barrier-associated responses (Mercer et al., 2025; Szabó et al., 2025; Linehan et al., 2018).

Ligand binding promotes AHR translocation to the nucleus, where it forms a complex with ARNT and regulates genes associated with epidermal differentiation, xenobiotic metabolism, immune responses, and barrier function. Experimental models show that skin commensals and microbiota-derived tryptophan metabolites can promote epidermal differentiation and barrier repair through AHR signaling. However, the response varies according to ligand identity, concentration, duration of exposure, and cell type. AHR activation cannot therefore be considered uniformly beneficial (Uberoi et al., 2021, 2025).

JAK/STAT signaling functions downstream of microbial recognition rather than as a direct PRR pathway. Cytokines induced after PRR activation, including IL-6, interferons, IL-4, IL-13, and IL-23, activate specific combinations of Janus kinases and STAT proteins in keratinocytes and resident immune cells. JAK/STAT-dependent transcription regulates antimicrobial responses, epidermal differentiation, leukocyte recruitment, and inflammatory cell programs. In human keratinocytes, exposure to Gram-negative anaerobes associated with hidradenitis suppurativa induced inflammatory signaling that was partly dependent on TLR4 and JAK/STAT activity. The findings support bacteria-induced activation of this pathway in an inflammatory disease model but do not establish a comparable mechanism in chronological skin aging (Williams et al., 2024).

PPAR signaling links lipid sensing with epidermal differentiation and inflammatory regulation. PPARα, PPARβ/δ, and PPARγ are lipid-activated nuclear receptors that regulate distinct aspects of keratinocyte differentiation, barrier recovery, lipid metabolism, and NF-κB- or AP-1-dependent inflammatory responses. In mice, PPARα deficiency altered cutaneous bacterial density and community composition and was associated with NOD2 signaling, oxidative stress, and abnormal keratinocyte differentiation. The findings indicate that host PPARα signaling can influence cutaneous microbial ecology and epidermal homeostasis. However, whether fatty acids or other lipid derivatives produced by the human skin microbiome regulate PPAR activity during chronological aging remains unknown (Blunder et al., 2026).

Dendritic cells integrate microbial antigens and keratinocyte-derived signals to coordinate adaptive immune activation. Antigen presentation through major histocompatibility complex pathways enables discrimination between commensal and pathogenic stimuli, shaping downstream T cell responses. Commensal microorganisms, particularly *Staphylococcus epidermidis*, induce antigen-specific T cell populations characterized by immunoregulatory and tissue-supportive functions rather than pro-inflammatory phenotypes. Resulting CD8+ T cell populations display combined antimicrobial and regulatory activity, supporting pathogen control while maintaining tolerance to resident microbiota (Li et al., 2024).

Microbiota-dependent signaling regulates adaptive immune polarization by balancing effector and regulatory pathways. Commensal-derived signals drive IL-17A production, contributing to antimicrobial defense, while regulatory T cell recruitment constrains excessive immune activation and maintains tolerance to resident microbial communities. Integration of microbial cues with host signaling pathways establishes a dynamic equilibrium in which effector responses are tightly controlled by regulatory mechanisms (Akuzum and Lee, 2022; Uberoi et al., 2021). Such interactions can be interpreted within the broader framework of host–microbiome coupling, in which microbial ecological stability, barrier integrity, and host immune regulation operate as interdependent components. Impairment of this coordination may lead to host–microbiome decoupling and promote mutually reinforcing cycles of dysbiosis, barrier dysfunction, and inflammatory activation (Bautista and López-Cortés, 2026b).

Bidirectional communication between the microbiota and the immune system contributes to cutaneous immune homeostasis. Immune-derived antimicrobial peptides and cytokines regulate microbial composition and restrict the overgrowth of potentially pathogenic species, whereas microbial ligands and metabolites influence immune-cell activation, epidermal differentiation, and barrier function. Disruption of host–microbiota signaling can alter microbial community structure and amplify inflammatory pathways, establishing a feedback loop between dysbiosis and immune activation (Cha et al., 2025; Elettrico et al., 2026). The clinical relevance of microbiome–immune crosstalk also extends to cutaneous oncology, where microbiome-associated mechanisms have been examined as potential modulators of antitumor immunity and therapeutic response in melanoma (Bautista et al., 2025).

Functional capacity cannot be determined reliably from genus-level abundance. Species within the same genus, and even strains within a single species, may differ in genes involved in lipid degradation, proteolysis, fermentation, antimicrobial activity, and adaptation to environmental stress. Gene detection by metagenomic sequencing identifies functional potential but does not establish transcription, protein activity, or production of a biologically relevant metabolite. Assigning microbial functions to cutaneous phenotypes consequently requires complementary metatranscriptomic, metaproteomic, or metabolomic evidence (Fernández-Carro et al., 2025; Baek et al., 2026).

## Microbiome and cutaneous aging biology

The skin microbiome changes with age in parallel with alterations in cutaneous physiology. Microbial communities contribute to barrier integrity, immune regulation, and tissue homeostasis. Age-associated differences in microbial composition have been linked to reduced sebaceous activity, altered hydration, immune senescence, and changes in skin surface properties (Li et al., 2025b; Taléns-Visconti et al., 2026). Host physiological changes may reshape microbial community structure and function, while microbial metabolites and ligands may influence barrier and immune responses.

Cutaneous aging can be examined within the Hallmarks of Aging framework. Relevant processes include cellular senescence, mitochondrial dysfunction, stem cell exhaustion, epigenetic alterations, chronic inflammation, and dysbiosis. Senescent cells accumulate in epidermal and dermal compartments and may develop a senescence-associated secretory phenotype (SASP) characterized by inflammatory mediators and matrix-remodeling proteins. Mitochondrial dysfunction, reduced stem cell function, and epigenetic alterations further impair cellular homeostasis, tissue renewal, barrier recovery, and extracellular matrix maintenance (López-Otín et al., 2023; Yu et al., 2025; He et al., 2025).

Links between the skin microbiome and these aging processes remain incompletely defined. Cellular senescence, impaired tissue renewal, barrier dysfunction, altered lipid secretion, and inflammatory signaling can modify the physicochemical conditions that structure microbial communities. Microbial ligands and metabolites may also influence inflammation, redox regulation, and epigenetic control through PRR–NF-κB/MAPK, NLRP3, AHR, and PPAR signaling. Current evidence does not establish that age-associated microbiome changes initiate cellular senescence, mitochondrial dysfunction, stem cell exhaustion, or epigenetic drift in human skin (Szabó et al., 2025; Wang et al., 2025; Bautista and López-Cortés, 2026c).

Age-associated microbiome remodeling occurs within a cutaneous environment marked by declining sebaceous activity, altered hydration and surface pH, impaired barrier recovery, immune senescence, and cumulative environmental exposure. Reported reductions in lipid-adapted microorganisms, including *Cutibacterium*, together with increased representation of *Corynebacterium* and *Streptococcus*, may therefore reflect underlying changes in skin physiology rather than autonomous taxonomic shifts. Genus-level differences cannot determine whether microbial metabolic activity is reduced, maintained through functional redundancy, or assumed by strains with different functional properties (Howard et al., 2022; Sun et al., 2024; Ratanapokasatit et al., 2022). Current cross-sectional evidence, however, does not establish whether microbial remodeling precedes, follows, or accompanies cutaneous aging.

Potential functional consequences include altered microbial processing of sebaceous lipids and amino acids. Reduced substrate availability may modify lipase and fermentation activity, affecting the production of free fatty acids, propionate, acetate, and other molecules involved in surface acidification, colonization resistance, and organization of the epidermal lipid barrier. Changes in microbial tryptophan metabolism may also alter the availability of indole-3-acetic acid, indole-3-aldehyde, and indole-3-pyruvate. Experimental models indicate that these metabolites activate AHR-dependent programs in keratinocytes and support epidermal differentiation and barrier repair. Whether their production or biological activity changes during chronological aging has not been established (Jung et al., 2024; Khalil and Kowalski, 2026; Chen et al., 2026).

Changes in microbial activity may influence inflammatory responses in aging skin. Microbial lipoproteins, lipoteichoic acids, lipopolysaccharides, and flagellin can activate pattern-recognition receptors expressed by keratinocytes and resident immune cells. Depending on the receptor, downstream pathways may involve MyD88–IRAK–TRAF6, IKK–NF-κB, JNK, and p38 MAPK; TLR4 can also activate TRIF-dependent responses. These pathways regulate the expression of cytokines, chemokines, antimicrobial peptides, and matrix metalloproteinases. Reduced production of barrier-supportive metabolites or prolonged exposure to inflammatory microbial ligands may contribute to increased IL-1β and IL-6 production in the context of immunosenescence and inflammaging. NLRP3 inflammasome activity and cytokine-dependent JAK/STAT signaling may further sustain inflammation, whereas AHR and PPAR pathways may link microbial metabolites with epidermal differentiation, lipid metabolism, and barrier repair. Evidence supports separate interactions between microbial signals and each of these pathways, but their coordinated involvement in age-associated microbiome remodeling has not been demonstrated in longitudinal human studies. It also remains unclear whether altered microbial activity contributes to inflammation during aging or develops in response to age-related changes in the skin environment (Bautista and López-Cortés, 2026c; Wang et al., 2026).

Inflammatory and metabolic changes may subsequently intersect with oxidative stress and extracellular matrix remodeling. In human epidermal keratinocytes, experimental activation of TLR2 and TLR5 induces MMP-1 and MMP-9 through NF-κB and JNK signaling, with p38 MAPK also contributing to MMP-9 regulation. Keratinocyte- and immune-cell-derived cytokines and oxidative mediators provide a possible paracrine route through which microbial sensing at the epidermal interface may alter fibroblast matrix turnover in the dermis. Sustained activation of these pathways could favor collagenase activity and reduce net collagen maintenance, although the complete sequence from age-associated dysbiosis to dermal matrix loss has not been demonstrated in humans. Ultraviolet radiation, pollution, and endogenous metabolic stress remain major independent drivers and may simultaneously alter the cutaneous environment, microbial activity, and extracellular matrix homeostasis (Wang et al., 2025; Li R. et al., 2026; Ren et al., 2024).

Direct evidence for microbial regulation of fibroblast biology remains limited but provides mechanistic support for specific microbial products. In murine fibroblasts and mouse skin, butyrate generated during *Staphylococcus epidermidis* fermentation increased type I collagen through FFAR2-dependent ERK phosphorylation. In human dermal fibroblasts exposed to ultraviolet radiation, lipoteichoic acid isolated from *Lactobacillus plantarum* reduced ROS generation, ERK and JNK activation, AP-1 and NF-κB activity, and MMP-1 expression, while increasing type I procollagen. These experiments show that defined microbial products can modify pathways involved in collagen synthesis and degradation. Their relevance to chronological aging remains uncertain because the studies used a defined fermentation substrate, murine models, or an isolated bacterial component rather than naturally occurring microbiome changes in aged human skin (Negari et al., 2021; Hong et al., 2015).

Barrier dysfunction can modify both the cutaneous environment and microbial activity. Increased transepidermal water loss, disrupted lipid organization, and delayed epidermal recovery alter the substrates and physicochemical conditions available to resident microorganisms. Changes in microbial production of organic acids, ceramide-related metabolites, and antimicrobial compounds may subsequently affect surface pH, colonization resistance, and barrier repair. A reciprocal interaction is therefore possible, with age-related changes in skin physiology reshaping microbial ecology and microbial functional changes influencing barrier recovery. Longitudinal human evidence is still insufficient to confirm such a feedback mechanism (Baker et al., 2023; Çetinarslan et al., 2023).

The combined effects of impaired barrier recovery, chronic inflammation, oxidative stress, and extracellular matrix deterioration contribute to wrinkles, reduced elasticity, fragility, and diminished tissue resilience. Facial microbiome studies have associated these phenotypes with age-dependent community changes, but taxonomic associations alone cannot identify the microbial pathways responsible for clinical variation. Integration of strain-resolved metagenomics with metabolite measurements and host molecular phenotyping will be required to determine whether specific microbial functions are associated with wrinkle formation, loss of elasticity, or impaired recovery after cutaneous injury (Khalid et al., 2022).

Age-associated microbiome remodeling is therefore better interpreted as a change in ecological and functional organization than as the replacement of individual taxa. Mechanistically relevant features include microbial lipid and amino acid metabolism, indole–AHR signaling, organic acid production, SCFA–FFAR2–ERK signaling, PRR-dependent activation of NF-κB and MAPK pathways, and paracrine regulation of fibroblast matrix turnover. Evidence supporting these mechanisms derives mainly from reconstructed epidermis, cultured keratinocytes and fibroblasts, gnotobiotic systems, and murine models. Determining whether the same pathways contribute to human cutaneous aging or primarily respond to changes in the cutaneous environment will require longitudinal, functionally resolved, and anatomically standardized studies (Swaney et al., 2025; Russo et al., 2023; Uberoi et al., 2025).

## Microbiome dysbiosis in dermatologic conditions

Alterations in the skin microbiome have been reported across several inflammatory skin disorders. Although microbial signatures differ among diseases, dysbiosis is commonly characterized by changes in community composition, shifts in microbial diversity, and disruption of the balance between resident commensals and opportunistic microorganisms. Acne, atopic dermatitis, and rosacea represent distinct examples of disease-associated microbial alterations, each exhibiting characteristic ecological profiles reproduced across independent studies (Piazzesi et al., 2024).

Disease-associated abundance patterns provide ecological context but do not fully explain microbial involvement in cutaneous inflammation. Biological effects depend on strain composition, expressed metabolic pathways, virulence-associated programs, intermicrobial competition, and the host pathways activated by microbial products. Acne, atopic dermatitis, and rosacea are therefore considered not only in relation to microbial abundance but also through strain-level properties and functional interactions with the cutaneous barrier and immune system (Khalil and Kowalski, 2026).

### Acne

Acne vulgaris is associated with alterations in the composition and strain-level distribution of microorganisms inhabiting sebaceous skin. Current evidence indicates that the presence of *Cutibacterium acnes* alone does not distinguish diseased from healthy skin, as the species constitutes a dominant member of the normal cutaneous microbiota. Instead, acne is more consistently associated with reduced strain diversity and enrichment of specific *C. acnes* lineages that differ from those commonly detected in healthy individuals (Podwojniak et al., 2025; Feidenhansl et al., 2024).

Strain-resolved sequencing studies have demonstrated distinct *C. acnes* phylotype distributions within acne lesions, supporting the view that microbial heterogeneity is altered during disease. Reduced diversity within *C. acnes* populations has been repeatedly reported in acne cohorts, whereas healthy skin generally contains a broader representation of strains. Such observations have shifted the interpretation of acne-associated dysbiosis from simple bacterial overabundance toward alterations in microbial community structure. Lineage-level differences may also have functional consequences. *C. acnes* strains vary in gene content, porphyrin production, lipase activity, biofilm formation, and capacity to activate inflammatory responses in keratinocytes and resident immune cells. Strain identity may therefore be more informative than total species abundance when evaluating microbial involvement in acne. However, the expression of such traits depends on follicular conditions and cannot be inferred solely from the detection of virulence- or metabolism-associated genes (Dreno et al., 2024; Feidenhansl et al., 2024).

Acne-associated dysfunction also involves interactions between *C. acnes, Staphylococcus* species, and other members of the follicular community. Intermicrobial competition, antimicrobial production, substrate utilization, and biofilm organization may alter the activity of acne-associated lineages without producing uniform changes in genus-level abundance. Community context is therefore important for determining whether a strain expresses inflammatory or potentially protective functions. Considerable variability remains across cohorts, anatomical sites, sampling strategies, and sequencing methodologies, limiting the identification of a universal functional signature (Podwojniak et al., 2025; Olszak et al., 2025).

### Atopic dermatitis

Atopic dermatitis exhibits one of the most reproducible microbiome signatures among inflammatory skin disorders. A consistent finding across studies is reduced microbial diversity accompanied by marked predominance of *Staphylococcus aureus*, particularly in lesional skin and during periods of increased disease activity (Huang et al., 2024).

Comparisons between healthy and affected skin have demonstrated that *S. aureus* frequently becomes the dominant bacterial species within eczematous lesions, while the abundance of several commensal microorganisms declines. The resulting ecological shift is accompanied by loss of microbial richness and reduced community complexity relative to healthy skin. Metagenomic analyses have further shown that greater *S. aureus* predominance is often observed in patients with more severe clinical manifestations, supporting an association between microbial imbalance and disease activity (Huang et al., 2024; Kim et al., 2025; John et al., 2026).

The biological relevance of *S. aureus* expansion extends beyond species abundance. Strains differ in quorum-sensing activity, toxin production, proteolytic capacity, adhesion, and other virulence-associated programs that can influence barrier disruption and inflammatory signaling. Loss of coagulase-negative staphylococci may further reduce antimicrobial competition and permit greater persistence or activity of pathogenic lineages. Disease severity may therefore depend on strain identity, expressed virulence pathways, surrounding community structure, and host barrier status rather than on *S. aureus* abundance alone. The temporal sequence between functional microbial changes and disease exacerbation nevertheless remains unresolved (Parlet et al., 2019; Dreno et al., 2024).

### Rosacea

Rosacea is associated with microbial alterations involving both cutaneous microorganisms and host inflammatory responses. Compared with acne and atopic dermatitis, reported microbial signatures are less uniform, although several taxa have been repeatedly implicated across independent studies (Lee et al., 2025; Kim et al., 2024).

Increased abundance of *Demodex folliculorum* represents one of the most frequently reported findings in rosacea. Elevated densities of the mite have been associated with shifts in local microbial communities and increased representation of microorganisms including *Bacillus oleronius* and *Staphylococcus epidermidis*. Alterations involving *Cutibacterium acnes* have also been reported, although observations vary across rosacea phenotypes and study populations (Asees et al., 2025).

The functional contribution of the rosacea-associated microbiome remains less clearly resolved. Increased *Demodex* density may modify the quantity and composition of microbial antigens and products encountered by keratinocytes and resident immune cells. Associated bacterial strains may further influence innate immune activity through surface molecules, enzymes, and other inflammatory signals. Available evidence therefore supports an interaction among microbial activity, barrier disturbance, vascular responses, and host susceptibility rather than a process driven by a single microorganism (Kim et al., 2024; Lee et al., 2025; Asees et al., 2025).

Across acne, atopic dermatitis, and rosacea, taxonomic findings provide ecological context but do not fully explain disease-associated microbial activity. Acne is more consistently linked to lineage-specific properties of *C. acnes* and interactions within the follicular community. Atopic dermatitis involves strain-dependent *S. aureus* virulence programs and reduced antimicrobial competition from resident commensals. Functional mechanisms in rosacea remain less defined but appear to involve interactions among microbial products, innate immune signaling, vascular responses, and host susceptibility. Strain identity, pathway activity, metabolite production, and host response therefore provide greater mechanistic resolution than genus- or species-level abundance alone (Sánchez-Pellicer et al., 2023; Zhu et al., 2023; Rušanac et al., 2025).

## Translational implications in dermatology and aesthetic medicine

### Skin aging interventions

In skin aging management, microbiome-oriented interventions are increasingly being incorporated into topical skincare strategies. Probiotic-, postbiotic-, and microbiome-supportive formulations have been evaluated for their effects on skin quality parameters, including hydration, elasticity, firmness, and wrinkle appearance. Several studies have reported improvements in these outcomes following the use of microbiome-targeted products, supporting their potential role as adjuncts to conventional anti-aging regimens. Interpretation of available findings remains cautious because studies differ substantially in formulation design, treatment duration, and clinical endpoints (Hong et al., 2025; da Silva et al., 2026).

Beyond direct skincare applications, the microbiome may also influence treatment outcomes in aesthetic dermatology. Available evidence suggests that microbial composition may contribute to variability in treatment response, recovery, and post-procedure outcomes, although clinically validated microbiome-based predictors remain unavailable. Greater understanding of microbiome-related variability may help refine patient selection and post-treatment management in future clinical practice, particularly as microbiome-informed approaches continue to be explored within personalized dermatology (Haykal et al., 2024; Haykal and Rocha, 2025).

### Aesthetic procedures

The relationship between the microbiome and treatment outcomes has gained attention in procedural dermatology. Many aesthetic interventions involve temporary disruption of the skin surface, followed by microbial recolonization during recovery. Variability in recolonization patterns may influence barrier restoration, skin tolerance, and post-procedure recovery. Although available evidence remains limited, understanding how microbial communities respond to dermatologic interventions may help refine treatment protocols and optimize post-procedure care (Haykal et al., 2024; Ellis et al., 2024).

Laser procedures represent one of the most extensively discussed examples of microbiome-procedure interactions. Both ablative and non-ablative laser modalities modify the cutaneous environment and may transiently alter microbial composition during healing. Available studies suggest that microbiome changes can occur after laser treatment, although the duration and clinical significance of such alterations remain incompletely characterized. Consequently, increasing attention has been directed toward post-laser strategies that support microbial recovery while maintaining procedural efficacy. Topical products containing probiotics or postbiotics have been proposed as potential adjuncts during the recovery phase (Haykal and Rocha, 2025; Ellis et al., 2024; Dinić et al., 2025).

Information regarding microbiome responses to chemical peels remains comparatively limited. Nevertheless, chemical resurfacing procedures involve controlled barrier disruption followed by microbial recolonization, making microbiome recovery a relevant consideration during post-procedure care. Similar considerations apply to microneedling. Clinical observations suggest that microneedling can be performed without evidence of persistent microbiome disruption while maintaining favorable tolerability profiles. Additional studies are needed to determine how different treatment parameters influence microbial recovery and long-term skin outcomes (Li et al., 2025a; Alqam et al., 2023).

### Injectables and biomaterials

Injectable procedures introduce distinct microbiome-related considerations. Dermal fillers and other implanted biomaterials have been associated with rare delayed complications in which microbial contamination and biofilm formation have been implicated. Biofilms may develop on implanted materials and have been reported in association with delayed nodules, chronic inflammatory reactions, abscess formation, and other late adverse events following filler placement. Recognition of these complications has reinforced the importance of appropriate patient selection, aseptic technique, and long-term clinical monitoring (Zhang et al., 2023; Baranska-Rybak et al., 2024).

Low-grade inflammatory reactions have also been described following filler administration. Delayed inflammatory responses may occur months after treatment and have been linked to multiple contributing factors, including biomaterial characteristics, host factors, and potential microbial involvement. Current evidence does not support a single unifying cause for all delayed reactions, highlighting the need for individualized evaluation and management. Continued investigation is required to clarify the contribution of microbial communities to these uncommon complications (Imen, 2023; Akyol et al., 2025).

### Microbiome-based therapies

Microbiome-based therapies represent an expanding area of translational research in dermatology and aesthetic medicine. Topical probiotics have received particular attention because of their potential to support skin quality and maintain microbial balance. Postbiotics offer an alternative approach that avoids challenges associated with the incorporation of live microorganisms while retaining desirable functional properties. In parallel, microbiome-targeted formulations containing probiotics, prebiotics, postbiotics, bacterial lysates, or microbial-derived ingredients are increasingly being incorporated into cosmetic and dermatologic products.

Live biotherapeutic products (LBPs) constitute a distinct category because they contain viable microorganisms developed for the prevention or treatment of disease. Clinical development in dermatology has focused mainly on atopic dermatitis. Topical application of *Staphylococcus hominis* A9 met the primary safety endpoint in a phase I trial and reduced *Staphylococcus aureus* abundance, although eczema severity did not differ significantly across the full study population. FB-401, a formulation containing three *Roseomonas mucosa* strains, showed an acceptable safety profile but was not superior to placebo in a multicenter phase II trial. The available trials indicate that short-term safety and changes in microbial abundance do not necessarily correspond to improvement in clinical severity (Jacobson et al., 2024; Wang et al., 2026).

Bacteriophage therapy provides a selective approach for reducing defined bacterial populations. A topical three-phage gel targeting *Cutibacterium acnes* was safe and well tolerated in a phase I cosmetic trial and reduced facial *C. acnes* burden. The study did not establish whether the reduction in bacterial abundance improved acne severity. Further development must account for variation in bacterial susceptibility, restricted host range, phage resistance, penetration into follicles and biofilms, and formulation stability (Golembo et al., 2022; Oh and Voigt, 2025).

Engineered commensal microorganisms are being investigated as local delivery systems for antigens, antimicrobial compounds, and defined microbial products. In murine melanoma models, *Staphylococcus epidermidis* engineered to express tumor antigens induced tumor-specific T-cell responses against local and metastatic lesions. Comparable clinical efficacy has not been demonstrated in humans. Genetic stability, persistence, horizontal gene transfer, microbial shedding, environmental release, and biological containment would require evaluation before clinical use (Oh and Voigt, 2025; Chen et al., 2023).

Microbiome-guided precision dermatology would integrate strain-level microbial profiles and functional measurements with anatomical site, sebum composition, surface pH, hydration, barrier integrity, inflammatory status, and previous treatment. Prospective validation would be required before such information could guide the selection of LBPs, bacteriophages, or other microbiome-directed interventions. Clinical implementation is limited by the absence of validated biomarkers, standardized sampling procedures, and prospective studies linking microbial profiles with treatment response or post-procedure recovery (Geusens and Haykal, 2025; Hutchinson et al., 2026).

Regulatory requirements depend on product composition, intended use, and therapeutic claims. Development of LBPs requires control of strain identity, purity, potency, viability, stability, and manufacturing consistency. Engineered microorganisms and bacteriophages may require additional genomic and environmental safety assessment. In the United States, products intended for therapeutic use are evaluated under investigational drug requirements that include chemistry, manufacturing, and control documentation. Cosmetic formulations may follow a different regulatory route depending on their intended use and stated claims (Dreher-Lesnick et al., 2017; Fukaya-Shiba et al., 2025).

Available evidence remains preliminary, and larger controlled clinical studies are needed to establish efficacy, standardize formulations, and define their role within routine aesthetic practice (Wang et al., 2025; Prajapati et al., 2025; Łetocha et al., 2025). At a broader translational level, frameworks for rational microbiome modulation and precision-health innovation provide a basis for moving from descriptive microbial profiles toward functionally informed interventions (Bautista and López-Cortés, 2026a). Application of these principles to dermatology will nevertheless require skin-specific validation of local ecological effects, formulation stability, barrier compatibility, clinical efficacy, and long-term safety.

## Methodological limitations and sources of heterogeneity

Interpretation of the relationship between the skin microbiome and cutaneous aging is constrained by marked methodological heterogeneity across studies. Differences in sampling procedures, sequencing strategies, contamination control, bioinformatic pipelines, and functional resolution complicate comparisons among cohorts and may contribute to inconsistent age-associated microbial patterns (Suri et al., 2025). Related problems have been documented in tumor microbiome studies, particularly platform-dependent variation, contamination of low-biomass samples, predominance of associative evidence, and limited integration of multi-omic data. Similar limitations across different microbiome fields suggest that they reflect broader methodological challenges in study design and microbial measurement rather than problems specific to skin microbiome research (Bautista and López-Cortés, 2026c).

Population characteristics further affect reported associations. Studies vary in participant selection, age-group definition, geographic setting, environmental exposure, cosmetic use, and hygiene practices, yet such variables are not consistently measured or included as covariates. Small cohorts and cross-sectional sampling also reduce statistical power, prevent evaluation of within-person microbial change, and increase susceptibility to unmeasured confounding. Associations attributed to chronological aging may therefore partly reflect cohort composition, lifestyle, environmental conditions, or analytical procedures. Greater methodological standardization and longitudinal sampling are required to distinguish reproducible age-related changes from study-specific variation (Ruuskanen et al., 2022; Knight et al., 2018).

A major challenge in skin microbiome research is the low microbial biomass characteristic of many cutaneous niches, particularly dry skin sites. Because microbial DNA is often present in limited quantities relative to host DNA, skin samples are especially vulnerable to contamination originating from collection devices, extraction kits, laboratory reagents, sample handling procedures, and sequencing workflows. In low-biomass samples, contaminant DNA may constitute a substantial proportion of the recovered signal and distort community composition if appropriate controls are not implemented. Recent methodological frameworks emphasize the importance of extraction blanks, negative controls, contamination-aware study design, and transparent reporting of contamination-removal procedures when investigating low-biomass microbial ecosystems (Santiago-Rodriguez et al., 2023; Plaza Oñate et al., 2025).

Differences in sequencing strategies represent another important source of heterogeneity. Most earlier studies relied on 16S rRNA gene sequencing, which remains useful for taxonomic profiling but is affected by primer-dependent amplification bias, variable taxonomic resolution, and limited functional characterization. In addition, 16S approaches are restricted to prokaryotic organisms and do not capture fungal, viral, or broader functional components of the skin microbiome. Shotgun metagenomic sequencing offers improved species- and strain-level resolution, permits analysis of non-bacterial taxa, and provides insight into microbial functional potential. However, shotgun approaches require greater sequencing depth and are challenged by high levels of host DNA contamination in skin samples. Comparative studies indicate that methodological choice can substantially influence diversity estimates and taxonomic composition, particularly in low-biomass skin environments, complicating direct comparison among studies (Eisenhofer et al., 2019; Markey et al., 2025).

Improved taxonomic resolution does not necessarily provide evidence of microbial activity. Shotgun metagenomics identifies genes and pathways encoded by a microbial community, but functional potential may differ from transcriptional activity, protein abundance, metabolic flux, and metabolite concentrations at the skin surface. Metatranscriptomics, metaproteomics, and metabolomics provide complementary information, although their application to skin remains constrained by low microbial biomass, high host-background signals, temporal variability, and incomplete microbial reference databases. Strain-resolved analysis is also required because lineages grouped within the same species-level classification may differ in metabolic capacity, antimicrobial activity, stress responses, and inflammatory potential. Functional claims should therefore distinguish between pathway detection, pathway expression, measured biochemical output, and demonstrated effects on the host (Jovel et al., 2022; Franzosa et al., 2015).

Anatomical variability introduces an additional layer of complexity. The skin consists of distinct ecological niches characterized by differences in moisture, sebum production, pH, temperature, oxygen availability, and microbial density. Sebaceous, moist, and dry regions harbor markedly different microbial communities, and microbial variation between body sites may exceed differences associated with chronological aging. Consequently, studies focusing on different anatomical locations often report distinct microbial profiles, making cross-study comparisons difficult. Findings derived from a specific skin site should therefore be interpreted within the context of local cutaneous microenvironmental conditions rather than generalized to the entire skin surface (Wang et al., 2025; Yadav and Bhambi, 2026).

Another important limitation is the restricted ability of current evidence to support causal inference. Most studies examining aging-associated microbiome alterations are observational and cross-sectional, identifying correlations rather than temporal or mechanistic relationships. Whether microbial changes contribute directly to age-related skin alterations, arise as a consequence of physiological aging, or reflect shared environmental and host-related factors remains uncertain. Longitudinal studies remain comparatively scarce, and experimental validation of proposed microbiome-mediated mechanisms remains insufficient. Many reported associations should therefore be regarded as hypothesis-generating rather than evidence of causality (Suri et al., 2025; Howard et al., 2022). Establishing causal relationships will require longitudinal designs, functional validation, and experimental approaches capable of distinguishing biological drivers from secondary microbial alterations (Ascandari et al., 2026).

The absence of methodological standardization remains a broader challenge for the field. Considerable variation exists in sampling procedures, storage conditions, DNA extraction methods, sequencing platforms, bioinformatic pipelines, reference databases, contamination-filtering strategies, and statistical analyses. Such differences may influence microbial diversity estimates, taxonomic assignments, and functional interpretations, even when similar biological samples are analyzed. Recent recommendations have emphasized standardized workflows, comprehensive metadata collection, contamination monitoring, and transparent reporting practices to improve reproducibility and facilitate comparisons across studies. Greater harmonization of laboratory, analytical, and reporting procedures will be necessary to establish robust and reproducible microbial signatures associated with cutaneous aging (Ruuskanen et al., 2022; Plaza Oñate et al., 2025; Mirzayi et al., 2021).

## Conclusions and future directions

Available evidence places the skin microbiome within the biological context of cutaneous aging. Age-associated changes in microbial composition, diversity, and functional potential have been reported across independent studies, although interpretation remains constrained by differences in cohort design, sampling site, sequencing methodology, and skin phenotyping. Current findings support an association between microbial ecology and aging-related skin characteristics, but do not establish a direct causal role for specific taxa or microbial pathways (Sun et al., 2024; Byrd et al., 2018; Swaney et al., 2025).

Further studies should determine whether microbial changes precede, follow, or accompany age-related alterations in skin physiology. Most available evidence is cross-sectional, limiting temporal interpretation. Longitudinal cohorts, controlled intervention studies, and human-relevant skin models are needed to evaluate whether microbial communities influence measurable aging phenotypes or primarily reflect changes in sebum production, pH, hydration, barrier integrity, immune function, and environmental exposure. Standardization and longitudinal study designs may also help distinguish persistent aging-associated microbial patterns from transient or site-specific variation (Suri et al., 2025; Wagner et al., 2024).

Research on age-associated changes in the skin microbiome requires resolution beyond taxonomic abundance. Studies should separate encoded functional potential from gene transcription, protein abundance, metabolite production, and host response. Shotgun metagenomics can resolve species, strains, and microbial pathways, whereas metatranscriptomic, metaproteomic, and metabolomic data are required to assess pathway activity and the presence of biologically relevant products. Linking these measurements with sebum composition, surface pH, hydration, barrier integrity, inflammatory signaling, oxidative stress, and extracellular matrix remodeling could identify microbial functions related to specific aging phenotypes. Longitudinal sampling with strain-level resolution is particularly important for separating persistent functional alterations from short-term ecological fluctuations (Fernández-Carro et al., 2025; Long et al., 2025; Elettrico et al., 2026).

Spatial microbiomics may clarify whether microbial taxa and their functions are restricted to follicles, sebaceous units, epidermal regions, or areas of barrier disruption, and whether their localization corresponds to specific host responses. Single-cell and single-nucleus sequencing may identify changes in keratinocytes, fibroblasts, sebocytes, and resident immune cells that are obscured in bulk tissue analyses (Yu et al., 2025; Lötstedt et al., 2024). Systems-biology approaches can integrate microbial activity with metabolites, skin physiology, environmental exposures, and clinical phenotypes to examine coordinated host–microbiome networks (Muller et al., 2024). Machine-learning methods may assist with multimodal data integration, feature selection, and prediction of aging phenotypes or treatment responses. Their use, however, requires control of confounding and batch effects, transparent model reporting, external validation, and experimental confirmation (Muller et al., 2024; Lan et al., 2024; Cheng et al., 2024).

Clinical translation requires a cautious and evidence-based framework. Microbiome-informed dermatology should not rely solely on microbial abundance profiles but on reproducible biomarkers linked to clearly defined clinical contexts. Integration of microbiomic, genetic, epigenetic, environmental, and clinical information may improve patient stratification and help explain variation in responses to skincare, dermatologic therapies, and aesthetic procedures (Geusens and Haykal, 2025; Bautista et al., 2026). Before clinical implementation, microbiome-informed strategies will require analytical validation, prospective evaluation, regulatory oversight, and evidence of added value beyond conventional clinical assessment (Madaan et al., 2024; Shen et al., 2023).

Age-related microbial changes should be interpreted within a bidirectional host–microbiome framework rather than as isolated taxonomic observations. Changes in skin physiology can alter microbial community structure and function, while microbial products may influence barrier integrity and immune responses. Current evidence does not establish whether microbial remodeling is a cause, consequence, or correlate of cutaneous aging. Determining temporal direction and functional relevance will require longitudinal human studies that integrate strain-resolved microbiome data with measurements of microbial activity, host molecular phenotypes, and clinical skin parameters (Suri et al., 2025; Li et al., 2020).
